# Integration of Tracking, Re-Identification, and Gesture Recognition for Facilitating Human–Robot Interaction

**DOI:** 10.3390/s24154850

**Published:** 2024-07-25

**Authors:** Sukhan Lee, Soojin Lee, Hyunwoo Park

**Affiliations:** 1 Department of Artificial Intelligence, Sungkyunkwan University, Suwon 16419, Republic of Korea; christie74@skku.edu; 2 Department of Electrical and Computer Engineering, Sungkyunkwan University, Suwon 16419, Republic of Korea; hwpark0104@skku.edu

**Keywords:** human–robot interaction, person tracking, person recognition, re-identification, gesture recognition

## Abstract

For successful human–robot collaboration, it is crucial to establish and sustain quality interaction between humans and robots, making it essential to facilitate human–robot interaction (HRI) effectively. The evolution of robot intelligence now enables robots to take a proactive role in initiating and sustaining HRI, thereby allowing humans to concentrate more on their primary tasks. In this paper, we introduce a system known as the Robot-Facilitated Interaction System (RFIS), where mobile robots are employed to perform identification, tracking, re-identification, and gesture recognition in an integrated framework to ensure anytime readiness for HRI. We implemented the RFIS on an autonomous mobile robot used for transporting a patient, to demonstrate proactive, real-time, and user-friendly interaction with a caretaker involved in monitoring and nursing the patient. In the implementation, we focused on the efficient and robust integration of various interaction facilitation modules within a real-time HRI system that operates in an edge computing environment. Experimental results show that the RFIS, as a comprehensive system integrating caretaker recognition, tracking, re-identification, and gesture recognition, can provide an overall high quality of interaction in HRI facilitation with average accuracies exceeding 90% during real-time operations at 5 FPS.

## 1. Introduction

Human–robot interaction (HRI) represents a significant technical thrust in robotics, aimed at maximizing robot serviceability for users. To date, advancements in HRI technologies have transformed the role of robots from passive tools to active collaborators with humans, capable of conducting proactive physical and social interactions. Consequently, there is a growing need for robots capable of physically and socially interacting with humans by accurately and reliably understanding human intentions and emotions, as well as social norms, in a natural setting, thus enabling mutual acceptance and trust. Various approaches to verbal, non-verbal, and multi-modal HRI, aimed at identifying human intent and emotion, have been proposed with the goal of fostering natural, robust, and user-friendly interaction.

However, despite the rapid evolution of technological advancement, the widespread application of HRI in intelligent robotic services has yet to be realized in practice. For instance, Lee et al. [1] proposed human–robot trust in HRI as a critical challenge for which transparent and effective communication strategies toward seamless collaboration and coexistence between humans and robots are essential. This may be due to that the success of real-world implementations of robotic services relies on various technologies in addition to an understanding of human intent and emotions. For instance, this requires accounting for (1) the robot’s proactive maintenance of readiness for interaction with the user at any time and place, for example, through the recognition and tracking of the user in a cluttered scene; (2) sufficient robustness and generalization in interaction implementations to handle variations, disturbances, and novelties in the interaction environment; (3) maintenance of a natural pace in interactions based on real-time execution of a system integrated with various technical components; and (4) optimal customization to the unique features associated with specific applications.

In this paper, we develop an integrated, real-time HRI system for a transport robot that carries a patient or a person needing assistance in a cluttered environment, in collaboration with caretakers [2]. Particularly, we emphasize the implementation of real-time caretaker recognition, re-identification, and tracking, along with caretaker gesture recognition, in a cluttered scene, enabling the robot to proactively maintain readiness for interaction with the caretaker anytime and anywhere. Furthermore, we aim to make the proposed robot–caretaker interaction system generalized and robust against variations, disturbances, and novelties in interaction environments. This contributes to reducing the caretaker’s burden in initiating interactions with the robot and enhances efficiency while conserving energy. During the system’s implementation, we explore deep learning approaches for person detection, recognition, re-identification, and gesture recognition to achieve efficient and robust system performance.

### 1.1. Related Work

Over the past decades, HRI-based frameworks have been implemented and exploited in unstructured environments such as homes and hospitals due to the broad range of issues HRI addresses. In this regard, we concentrate on a vision-based HRI system that encompasses person detection, recognition, tracking, re-identification, and gesture recognition, aiming to achieve natural and sociable HRI. A considerable number of research studies [3,4,5,6,7,8,9,10,11,12,13,14,15,16,17,18,19,20,21,22,23,24,25,26,27,28,29,30,31,32,33] in the literature have investigated individual vision-based HRI components, including person detection, recognition, re-identification, and gesture recognition. Nevertheless, there has been scarce reporting on HRI systems that integrate various vision-based modules promoting natural and sociable HRI. Additionally, the recent surge in human–robot collaboration within smart manufacturing and the expansion of service robotics across domestic and professional settings have propelled the development of comprehensive HRI systems to meet the needs for natural and sociable interactions. For example, Sanjeewa et al. [3] developed a human–mobile robot interaction system designed to deliver objects to elderly or disabled individuals through a two-step gesture recognition process. An HRI system tailored for the elderly was crafted by Zhao et al. [4], incorporating a skeleton-based gesture recognition system. Furthermore, Liu et al. [5] introduced a system enabling real-time gesture-based communication with individuals outdoors for rescue purposes. Rollo et al. [6] presented a framework that incorporates person detection, segmentation, re-identification, and gesture recognition, utilizing RGB-D data for the real-time navigation of mobile robots. Additionally, Müller et al. [7] engineered a system that facilitates human detection, recognition, tracking, and re-identification for user-centric navigation such as robot-guided movement.

#### 1.1.1. Person Detection and Tracking

With advancements in deep learning, the technology for person detection in video frames has evolved to enable fast and robust person detection in real time. Popular object detection platforms such as Faster RCNN [8], various versions of YOLO [9], and SSD [10] have become available, among others [11,12,13]. Lee et al. [14] introduced a deep learning approach for detecting vehicles and pedestrians from CCTV footage utilizing YOLO as the foundational network and CNN for transfer learning. Similarly, Amir et al. [15] developed a detection method for individuals based on YOLO and CNN, employing more precise information categories such as stick/clutch and wheelchair/walk for enhanced accuracy.

The challenge of person tracking in crowded environments continues to attract attention from the mobile robotics and visual surveillance sectors. Tracking is often performed by predicting the trajectory of a detected person based on motion estimation. Managing the potential disappearance of a person being tracked in the crowd, whether temporarily or over an extended period, remains a critical challenge, potentially necessitating a re-identification process. In this context, Fernando et al. [16] proposed a deep learning-based multi-person localization and tracking approach, employing GAN for person localization and integrating a trajectory prediction scheme for both short- and long-term scenarios, along with cost-effective data association for tracking. Previously, Choi [17] introduced a multi-target tracking algorithm that leverages an aggregated local flow descriptor to provide a reliable affinity measure for linking temporarily separated detections and ensuring efficient and accurate data association between targets and detections in multi-target tracking scenarios. Additionally, Manzoor et al. [18] developed a system that uses edge devices to facilitate real-time person detection and tracking; based on tracking data, a mobile robot can follow a person. This tracking system merges deep learning with metric learning to provide robust tracking capabilities for various views of a person, including front, side, and back.

#### 1.1.2. Person Recognition and Re-Identification

Automatic recognition and re-identification of a person in natural and crowded scenes, based on video images, have captured the interest of the surveillance and robotic service sectors. The principal hurdles in person recognition and re-identification involve overcoming the technical challenges posed by variations in orientation, size, occlusion, and illumination of individuals appearing in video images. In the arena of face recognition, deep learning strategies, including DeepFace and DeepID, have become predominant, epitomizing the state of the art as highly evolved technologies [19]. To surmount the aforementioned challenges, methods like CNN, auto-encoder, and GAN-based approaches focusing on face normalization, super-resolution, and transfer learning utilizing a robust backbone network have been crafted for real-world deployment. Sohail et al. [20] introduced a deep learning system that proficiently recognizes a person despite various facial poses by adapting a segment of the YOLO network specifically for face recognition. Furthermore, Condés et al. [21] deployed RGB-D sensors on a mobile robot to detect and identify persons using FaceNet [22]. Subsequently, the robot tracks the identified person through optical tracking based on the detection outcomes.

Person re-identification typically relies on the matching of features extracted from pairs of detected persons, where the methodology of matching is pivotal as appearances of the same individual might differ due to changes in location and pose. The challenges of deep learning-based re-identification have been tackled through enhancements in feature representation learning, deep metric learning, and ranking optimization; for further information, refer to [23,24]. Wang et al. [25] unveiled a method for spatial–temporal person re-identification that harnesses a visual feature stream with spatial–temporal constraints, extracting visual features via the ResNet backbone network. Moreover, Rollo et al. [26] developed a system for re-identification using features derived from a detection network, enabling the recognition of a target person in complex settings like shopping malls and accommodating variations in the target’s appearance and visual occlusions. He et al. [27] presented a Dense Interaction Learning (DIL) method that leverages temporal information in video sequences to address the challenge of re-identification. Features are extracted from each frame, and rich information is learned from interactions between neighboring frames and combined into a unified representation for the final decision of re-identification. They represent a novel approach that overcomes the limitations of existing methods.

#### 1.1.3. Gesture Recognition

For gesture recognition, it is necessary to extract a person and his/her body parts from a series of image frames, based on which the person’s motion can be modeled. Pradyumna et al. [28] recognized gestures through a deep learning-based multi-channel approach, where a global channel looks for gross motion across the entire sequence of video images while a focused channel detects the motion of each hand. To further enhance the accuracy, Liang et al. [29] employed a combination of 3DCNN and bidirectional long-short-term memory network (LSTM) to extract spatiotemporal features for gesture recognition. Muneer et al. [30] introduced an approach for recognizing hand gestures using 3DCNN architecture to learn spatiotemporal features, employing transfer learning to address the scarcity of labeled hand gesture datasets. Dadashzadeh et al. [31] utilized semantic segmentation to identify hand gestures based on a dual-stream convolutional neural network (CNN) that integrates data from the hand region’s red-green-blue color channels and segmented images. Yu, J. [32] extracted spatial and temporal features using a video frame and optical flow frame, thereafter employing a dual-channel 2D CNN model to identify hand gestures through feature fusion. Zhu et al. [33] proposed an Action Machine which involves action recognition of 27 action classes based on RGB images only which obtained 92.5% accuracy on the UTD-MHAD [34] dataset. They used CNN for the single-frame feature extraction and optical flow to capture the temporal features from sequential frames. Despite advancements, gesture recognition continues to face significant technical challenges in achieving sufficient generalization power to handle a broad range of appearance variations and occlusions.

### 1.2. Problem Statement and Contribution

The problem we aim to address in this paper is how to implement a natural and real-time HRI system in a cluttered environment, specifically for interactions between a transport robot and a caretaker. We emphasize the integration of various lightly configured deep learning networks to effectively carry out caretaker recognition, tracking, re-identification, and gesture recognition in real time while maintaining accuracy sufficient for real-world applications. To achieve the desired robustness and generalization power, the integrated system should effectively handle disturbances, variations, and novelties occurring in interaction environments. Ultimately, we intend for the integrated system to enable the robot to proactively maintain readiness for anytime interaction while achieving sociable HRI based on gesture-based interaction. In this paper, we demonstrate that a seamless integration of tracking and re-identification can be achieved for high performance in both caretaker identification and gesture recognition, enabling robots to remain prepared for interactions with a caretaker.

## 2. Overview of the Proposed System

**Figure 1:**
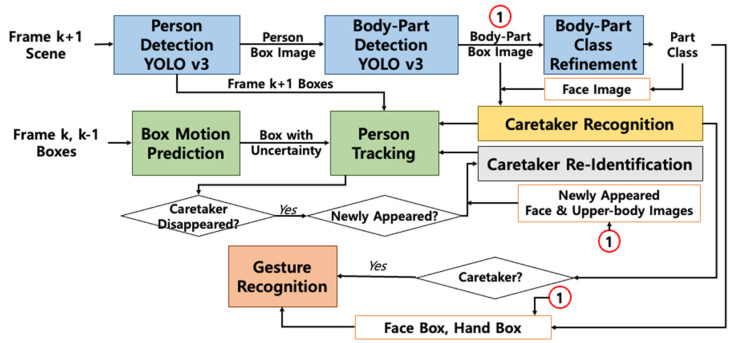
Overall flow architecture of the proposed Robot-Facilitated Interaction System (RFIS). Note that the number 1 in the red circle indicates the cropped body-part image sets detected from the cascaded YOLOv3.

Figure 1 illustrates the overall system flow of the proposed Robot-Facilitated Interaction System (RFIS). The system is composed of four major components: (1) the person and body-part detection process (in blue), (2) the person tracking process (in green), (3) the caretaker recognition and re-identification process (in yellow and gray), and (4) the caretaker gesture recognition process (in orange). Initially, persons and their body parts appearing in a scene are detected in each frame utilizing a cascaded configuration of YOLOv3 [35], which identifies various body parts such as the head/face, upper body, and hands. To enhance body-part labeling accuracy, an image-based deep body-part classifier is integrated with the cascaded YOLOv3 to refine body-part label classification. Subsequently, the detected persons and their body parts are tracked across a sequence of image frames based on the fusion of detected boxes from frame k + 1 with predicted boxes from frames k and k − 1, utilizing motion-based filtering techniques.

For effective facilitation of interactions with caretakers, the robot must be capable of recognizing caretakers as they enter the scene and continue tracking them, employing re-identification as needed to handle disappearances and reappearances. The caretaker recognition process begins either with the robot’s initial operation or whenever a new person emerges in the scene. Meanwhile, the caretaker re-identification process is triggered at each keyframe during person tracking to detect the caretaker’s disappearance and reappearance. Caretaker recognition employs facial images captured by the person detection network and analyzed using EfficientNet [36], while caretaker re-identification calculates the conditional probability that candidate facial and upper-body images, obtained from person and body-part detection networks, indeed represent the caretaker based on comparison with verified caretaker images. The recognized and tracked caretaker, along with his/her body parts, are then processed through an LSTM-based gesture recognition network to identify the gestures intended for the robot.

## 3. Person and Body-Part Detection and Classification

**Figure 2:**
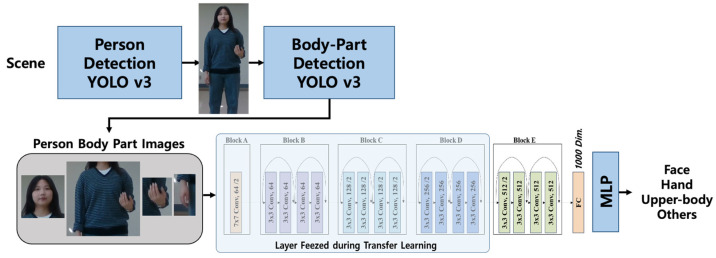
The proposed person and body-part detection and classification network is configured with the cascaded YOLOv3 and ResNet18-based transfer learning for feature embedding and classification.

Figure 2 illustrates the flow of the proposed deep person and body-part detection and classification network. The box image of a person detected in a scene by the initial YOLOv3 is cropped as the input for the subsequent YOLOv3, which focuses on detecting body parts such as the head, upper body, and right/left hand of the person. To minimize the omission of any body parts in detection, the non-maximum suppression threshold of the second YOLOv3 is adjusted, despite this adjustment causing a reduction in precision and labeling accuracy of the detected body parts. To counteract the decline in detection precision and labeling accuracy resulting from the reduced threshold, the box images identified by the second YOLOv3 are reclassified using a ResNet18-based image classifier, which is trained under a transfer learning framework. Specifically, images of the body parts identified by the second YOLOv3 are cropped and fed into the ResNet18 classifier for final body-part decision-making. For generating feature embeddings from these cropped body-part images, the ResNet18 model, initially trained with ImageNet datasets, is further refined with a custom body-part dataset via transfer learning. In this process, the pre-trained ResNet18 is frozen up to the fourth residual block, while re-training is conducted on the blocks above the fifth residual block using the custom body-part training dataset. The person and body-part box images, along with their labels, finalized by the cascaded YOLOv3 and associated body-part image classifier, are subsequently linked to the ensuing tracking, caretaker recognition, and re-identification processes described in the subsequent sections.

Note that the proposed RFIS needs to be mounted on an edge computing device (Jetson Xavier AGX, NVIDIA Corporation, Santa Clara, CA, USA) to perform caretaker recognition, re-identification, and gesture recognition in real time. Therefore, when choosing a deep learning model, the performance was taken into consideration in terms of the trade-off between the accuracy and the number of parameters that suit the required real-time processing of RFIS with Jetson Xavier AGX. Among the three versions of YOLOv3, v4, and v5, YOLOv3 is selected based on its processing speed with Jetson Xavier AGX [37] to maximally shorten the time required for person detection so that it does not burden the overall processing speed of RFIS.

## 4. Person Tracking

Persons identified in video sequences are subject to real-time tracking. The main aim of this process is to monitor the caretaker across different frames while noting his/her disappearance and reappearance. To accomplish this, person tracking operations are executed along with the video frames, during which both the disappearance of one person and the appearance of another are identified. The outlined process for person tracking encompasses the following stages: (1) Utilizing the movements of the person boxes depicted at frame k and frame k − 1, predictions are made regarding the location, size, and shape of person boxes for the subsequent frame k + 1. The movements of the person are derived from the temporal alterations in the position, size, and shape of person boxes throughout the video frames. (2) The person boxes recognized at frame k + 1 are subsequently merged with the person boxes predicted from frame k to determine the final location, size, and shape at frame k + 1. For predicting the location, size, and shape at frame k + 1, the motion trajectories of the 3D boxes and their uncertainties at frame k are calculated based on the temporal changes from frame k − 1 to frame k. For example, if a box’s motion is discerned to be moving left and getting closer, the predicted box will be positioned to the left and enlarged accordingly. Accounting for uncertainties in the estimated box motion, the location, size, and shape of the predicted person boxes are probabilistically adjusted, which is considered during the fusion of detected and predicted boxes at frame k + 1. Notably, to minimize tracking errors due to switching, the uncertainty expansion for size and shape is limited to the width direction, acknowledging the general horizontal movement of humans within an image plane. This procedure of merging detected and predicted boxes at frame k + 1 is referred to as fusion-based person tracking.

**Figure 3:**
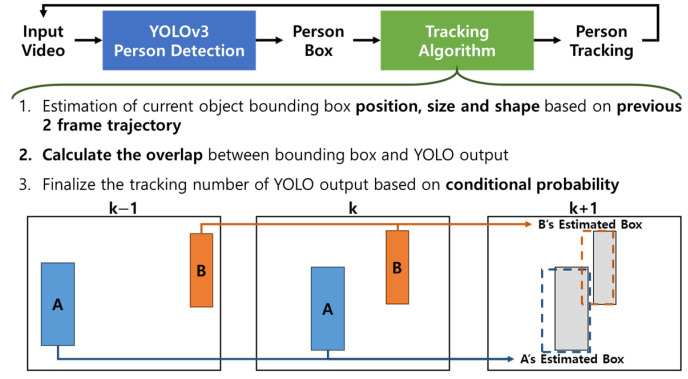
Schematic description of the proposed person/body-part tracking process with person prediction and fusion.

The proposed fusion-based person tracking method provides a systematic approach for the automatic identification and tracking of persons newly appearing in a scene, as well as for the re-identification and re-tracking of a person who reappears. Figure 3 schematically depicts the process of this fusion-based person tracking, which includes predicting person boxes from cascaded YOLOv3 at frame k + 1 and fusing the detected and predicted person boxes at the same frame. As detailed earlier, the predicted person boxes for frame k + 1 are derived by linearly extrapolating the changes in location, size, and shape of the person boxes from frame k − 1 to frame k. The fusion process at frame k + 1 begins by pairing matching detected and predicted boxes, if any, which track the same individual across the video frames. Initially, for each detected box, candidate-predicted boxes that meet a proximity threshold are selected for potential matching, followed by computing probabilistic matching scores based on similarities in box positions, sizes, shapes, and probabilities associated with box labels, while considering the uncertainties of the predicted box characteristics. If a detected box at frame k + 1 lacks a counterpart among the candidate-predicted boxes, it is labeled as newly appeared in the scene. Conversely, if a predicted box at frame k + 1 has no matching detected box, it is considered to have disappeared. The proposed person tracking based on motion-based box prediction is intended to make person tracking highly efficient, say, about four times faster than the state-of-the-art approach, BoostTrack [38]. To enhance tracking robustness, persons detected with high uncertainty are monitored for several additional video frames until these uncertainties are resolved. This advanced fusion-based process seamlessly manages the appearance and disappearance of persons, facilitating the recognition, tracking, and re-identification of the caretaker as the primary tracking target. For further details on this tracking method, refer to Algorithm 1.
**Algorithm 1.** Person Tracking AlgorithmInput: Individual boxes at frame k − 1 and k, YOLOv3 boxes at frame k + 1 Require: delta t = 30, rho = 0.04, iou_threshold = 0.4, T = 500**1:** *for i* in frame k − 1 and k boxes: **2: (frame k + 1 box linear estimation)**
    delta X, delta Y, delta W, delta H = (frame k − 1’s *i*-th box − frame k’s *i*-th box) **3: (Uncertainty Box)**
$O_{i}$    $O_{i}$ = [delta X, delta Y, delta t ∗ rho ∗ delta W, delta t ∗ rho ∗ delta H] **4: (Box Overlap)**
    *for j* in YOLOv3 boxes:        $Y_{j}$ = YOLOv3 *j*-th box at frame k + 1        Box overlap ratio between YOLOv3 boxes at frame k + 1 and Uncertainty Box **5: (Distance between**
$O_{i}$
**and**
$Y_{j}$**)**        *if* Box Overlap Ratio > iou_threshold:           distance = $\sqrt{0.3{\left(O_{i}c-Y_{j}c\right)}^{2}+0.4{\left(O_{i}w-Y_{j}w\right)}^{2}+0.4{\left(O_{i}h-Y_{j}h\right)}^{2}}$ **6: (Conditional Probability)**
        *for k* in *N*:         # *N* is the total number of line 5 result           $P\left(\frac{Y_{k}}{O_{i}\neq Y_{k}}\right)=1-e^{-\left(\frac{distance_{k}}{T}\right)}$: Negative Conditional Probability           $P\left(\frac{Y_{k}}{O_{i}=Y_{k}}\right)=e^{-\left(\frac{distance_{k}}{T}\right)}$: Positive Conditional Probability           *for n* in *N*              *if n*
≠
*k*:              $P\left(\frac{Y_{n}}{O_{i}\neq Y_{k}}\right)*=e^{-\left(\frac{distance_{n}}{T}\right)}$: Positive Conditional Probability              $P\left(\frac{Y_{n}}{O_{i}=Y_{k}}\right)*={1-e}^{-\left(\frac{distance_{n}}{T}\right)}$: Negative Conditional Probability              $P\left(\frac{O_{i}=Y_{k}}{Y_{1},Y_{2},\ldots ,Y_{N}}\right)=\frac{1}{1+\frac{P\left(\frac{Y_{k}}{O_{i}\neq Y_{k}}\right)P\left(\frac{Y_{N}}{O_{i}\neq Y_{k}}\right)}{P\left(\frac{Y_{k}}{O_{i}=Y_{k}}\right)P\left(\frac{Y_{N}}{O_{i}=Y_{k}}\right)}}$ **7: (Assign Tracking Number)**    The max value of Conditional Probability means that $Y_{k}$ is the same person as $O_{i}$.    $O_{i}$ trackingID = argmax($P\left(\frac{O_{i}=Y_{1}}{Y_{1},Y_{2},\ldots ,Y_{N}}\right),P\left(\frac{O_{i}=Y_{2}}{Y_{1},Y_{2},\ldots ,Y_{N}}\right),\ldots ,P\left(\frac{O_{i}=Y_{N}}{Y_{1},Y_{2},\ldots ,Y_{N}}\right)$)

## 5. Caretaker Recognition and Re-Identification

Automatic recognition and tracking of the caretaker in a scene represent one of the key tasks for the robotic facilitation of HRI, which requires monitoring the appearance, disappearance, and reappearance of the caretaker. To this end, the proposed RFIS checks video frames if the caretaker is identified in a scene or is lost in tracking, and if any newly appeared person in a scene is the caretaker in case the caretaker is lost in tracking.

### 5.1. Caretaker Recognition

Caretaker recognition is based on the facial image boxes of the persons detected as newly appearing when the caretaker is not present in a scene. The newly appeared facial image boxes are cropped from the output of the second YOLOv3 and up-sampled to input to EfficientNet for caretaker recognition. Note that, since a caretaker may change his/her outfit daily, we opt to rely on facial images for caretaker recognition instead of including other body-part images. For caretaker recognition, EfficientNet-B0 was employed as it is most light in terms of the number of parameters. In addition, we applied transfer learning to the full layers of a pre-trained EfficientNet-B0 in such a way as to optimize the caretaker recognition. On the other hand, in the case of the caretaker re-identification for which the outfit of the caretaker is surely maintained, both facial images and upper-body images are adopted for caretaker recognition.

### 5.2. Caretaker Re-Identification

**Figure 4:**
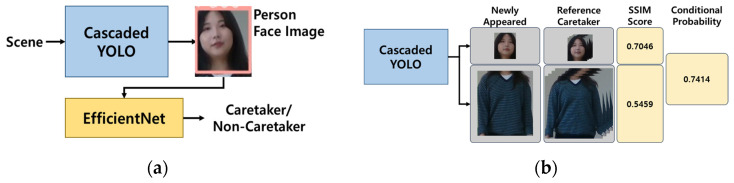
The schematic flow of the proposed caretaker recognition and re-identification process particularly highlights the SSIM-based re-identification of the caretaker: (**a**) the caretaker recognition network based on EfficientNet. (**b**) the caretaker re-identification based on SSIM of the face and upper body images.

Re-identification of the caretaker is crucial due to the potential disappearance and subsequent reappearance during tracking in crowded environments. This process is initiated when the caretaker box disappears for several video frames, during which the tracking remains intact until the probability of disappearance reaches a high threshold. Once the disappearance is confirmed, the re-identification protocol is activated to scrutinize newly appeared person boxes to detect the caretaker’s reappearance. The approach relies on measuring the similarity in facial and upper-body images between the previously detected caretaker box and the new candidate boxes, as depicted in Figure 4b. Specifically, reidentification involves computing the conditional probability that a newly appeared candidate box is the caretaker box, based on the similarity measure between them. To assess this similarity, the Structural Similarity Index Measure (SSIM) [39] is employed between the two images. The conditional probability for a candidate box being the caretaker is then derived using two similarity measures from the facial and upper-body images, as outlined in Equation (1).

(1) $$P\left(\frac{B_{j}=Caretaker}{{\left\{S_{f},S_{u}\right\}}^{B_{j}}}\right)=\frac{1}{1+\frac{P\left(\frac{{\left\{S_{f}\right\}}^{B_{j}}}{B_{j}\neq Caretaker}\right)P\left(\frac{{\left\{S_{u}\right\}}^{B_{j}}}{B_{j}\neq Caretaker}\right)P(B_{j}\neq Caretaker)}{P\left(\frac{{\left\{S_{f}\right\}}^{B_{j}}}{B_{j}=Caretaker}\right)P\left(\frac{{\left\{S_{u}\right\}}^{B_{j}}}{B_{j}=Caretaker}\right)P(B_{j}=Caretaker)}}$$

In Equation (1), ${\left\{S_{f}\right\}}^{B_{j}}$ and ${\left\{S_{u}\right\}}^{B_{j}}$ represent the similarity measures for facial and upper-body images, respectively, from the candidate box $B_{j}$ compared to the caretaker box. In our experiments, these similarity measures, ${\left\{S_{f}\right\}}^{B_{j}}$ and ${\left\{S_{u}\right\}}^{B_{j}}$, are directly used as the conditional probabilities, $P\left(\frac{{\left\{S_{f}\right\}}^{B_{j}}}{B_{j}=Caretaker}\right)$ and $P\left(\frac{{\left\{S_{u}\right\}}^{B_{j}}}{B_{j}=Caretaker}\right)$, respectively. For candidates likely not being caretakers, a default probability of 0.5 is assigned to both $P\left(\frac{{\left\{S_{f}\right\}}^{B_{j}}}{B_{j}\neq Caretaker}\right)$ and $P\left(\frac{{\left\{S_{u}\right\}}^{B_{j}}}{B_{j}\neq Caretaker}\right)$. The prior probability, $P\left(B_{j}=Caretaker\right)$, is determined by the effectiveness of the tracking in predicting the caretaker box; if less effective, a default value of 0.5 is assigned. The re-identification decision for the caretaker is made when the conditional probability of a candidate box surpasses a predefined threshold.

To utilize the proposed caretaker re-identification approach within the integrated RFIS system, we applied supplementary algorithms to ensure effective re-identification across contiguous video sequences. In scenarios where re-identification relies solely on a single frame, the error probability significantly increases due to limited evidence available for assessment. To enhance the reliability of this evidence, we instituted additional prerequisites, such as specific timing for collecting images of the caretaker’s head and upper body along with those of new candidates. For optimal results, it is crucial that the images being compared are captured under comparable conditions, fostering the most effective SSIM outcomes. Discrepancies in perspectives, such as comparing a side view with a front view, can lead to notably lower SSIM values, even if the individuals in both images are identical. In our approach, we accumulate 30 images of both the caretaker’s face and upper body from the initial moment of identification. This collection provides a diverse set of 30 facial and upper body images. When a new candidate appears after the caretaker has exited the scene, it is essential to promptly gather images of any newly surfaced individuals. Each image, capturing both the face and upper body, is taken over 40 frames beginning from their appearance. Subsequently, SSIM measurements of both the caretaker and the new candidate’s facial and upper-body images are conducted to compute the re-identification probability using Equation (1). The highest of the 30 probabilities is then selected as the definitive similarity score between the candidate and the caretaker. A re-identification probability of 0.7 or higher confirms the candidate’s identity as the caretaker, allowing gesture recognition to proceed without necessitating further recognition processes.

## 6. Caretaker Gesture Recognition

**Figure 5:**
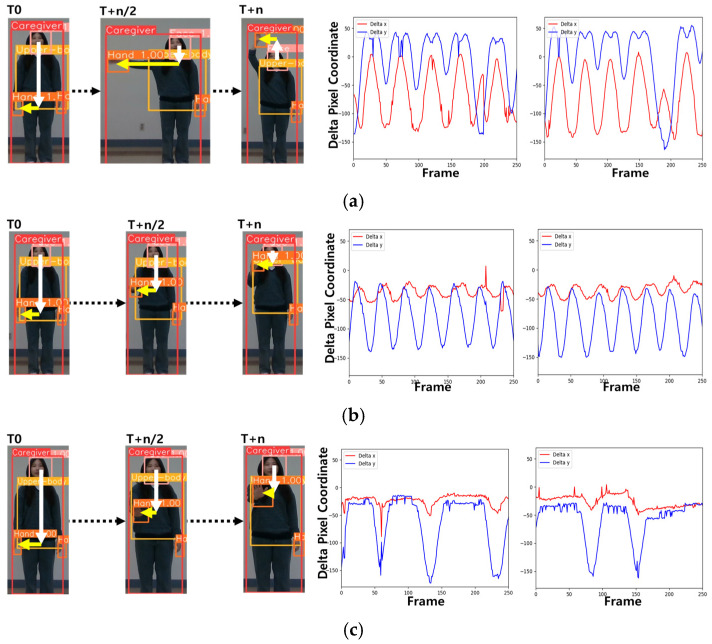

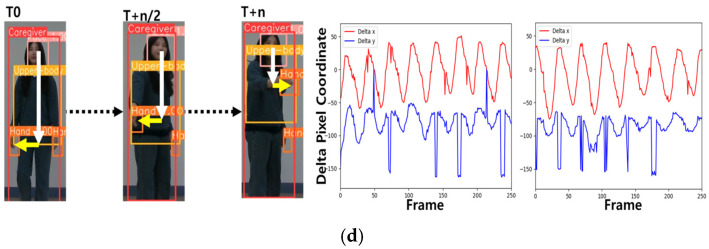
Four categories of pre-defined gestures to communicate with robots: (**a**) “Here I am”, (**b**) “Stay there”, (**c**) “Come here”, and (**d**) “Follow me”. Note that in each figure, the white and yellow arrows indicate the pixel coordinates from the center of the face box to the center of the hand box.

While tracking the caretaker, RFIS captures a sequence of the caretaker’s body parts, specifically focusing on the upper body and hands throughout the progression of video frames. This process is used to determine whether the caretaker is making a gesture intended for the robot, and if so, recognizes the gesture. RFIS pinpoints the intended gesture based on a sequence of body-part movements recorded sequentially during a pre-defined interval of video frames. To facilitate this, a series of gestures are pre-defined for the system to learn beforehand. Figure 5 delineates four categories of these pre-prepared gestures: “Here I am” (a), “Come here” (b), “Stay there” (c), and “Follow me” (d). Gestures not aligning with these are categorized as “No gesture”. For gesture recognition, we depicted a gesture as a trajectory of the poses of both hands relative to the center of the face box with reference to the pixel coordinate. To define these relative poses, we normalized the detected caretaker box to 256 × 256 in size such that the relative pose between face and hand becomes stabilized. Examining changes in delta x (red) and delta y (blue) across frames during the execution of pre-defined gestures shows notable differences; for instance, “Here I am” (a) exhibits a greater amplitude on the y-axis compared to the x-axis and sustains a longer gesture period than “Come here” (b) and “Follow me” (d) with higher amplitude. In contrast, “Come here” (b) demonstrates a greater emphasis on the y-axis than the x-axis, with smaller variations in delta x and delta y. “Follow me” (d) shares a similar gesture cycle with “Come here” (b) yet exhibits variations in delta x and delta y across different poses. “Stay there” (c) uniquely involves the hand rising and then maintaining a fixed position for a duration, distinctly affecting the behavior of the signal.

**Figure 6:**
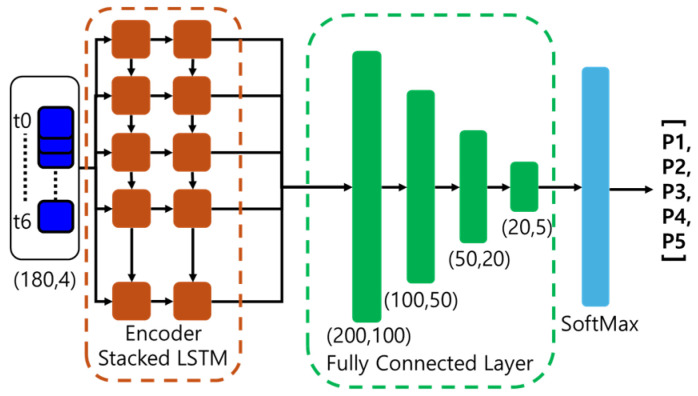
The proposed stacked LSTM architecture for gesture recognition.

Ultimately, hand pose class labels from 180 (6 s) consecutive video frames are fed into a stacked LSTM to encode the feature embedding, as depicted in Figure 6. This encoding is then processed through a fully connected network to classify the input into one of the five gesture categories, including “No gesture”.

## 7. Experimental Results 

To demonstrate the proposed robotic facilitation of HRI, we integrated the person/body-part detection and classification, person tracking, caretaker re-identification, and caretaker gesture recognition developed individually into a real-time RFIS. The integrated RFIS keeps track of the caretaker and recognizes his/her gestures in real time based on the RGB-D data captured by the Intel RealSense D455 as its input. Despite its limited computational power, we adopted the Nvidia Jetson AGX Xavier, an edge computing device, for implementing RFIS, particularly for processing the deep learning networks involved. Note that an edge computing device was adopted for processing to ensure that the entire system can be easily worn and carried by a user. Given the limited computational power, we managed to process the integrated RFIS on the Jetson AGX Xavier with a processing speed of approximately 0.2097 s per frame. In the experiment, we evaluated the end-to-end performance of RFIS in terms of identifying, tracking, and re-identifying the caretaker as well as recognizing his/her gestures, if any, such that the quality of interaction between the robot and the caretaker provided by the proposed RFIS was adequately assessed. 

**Figure 7:**
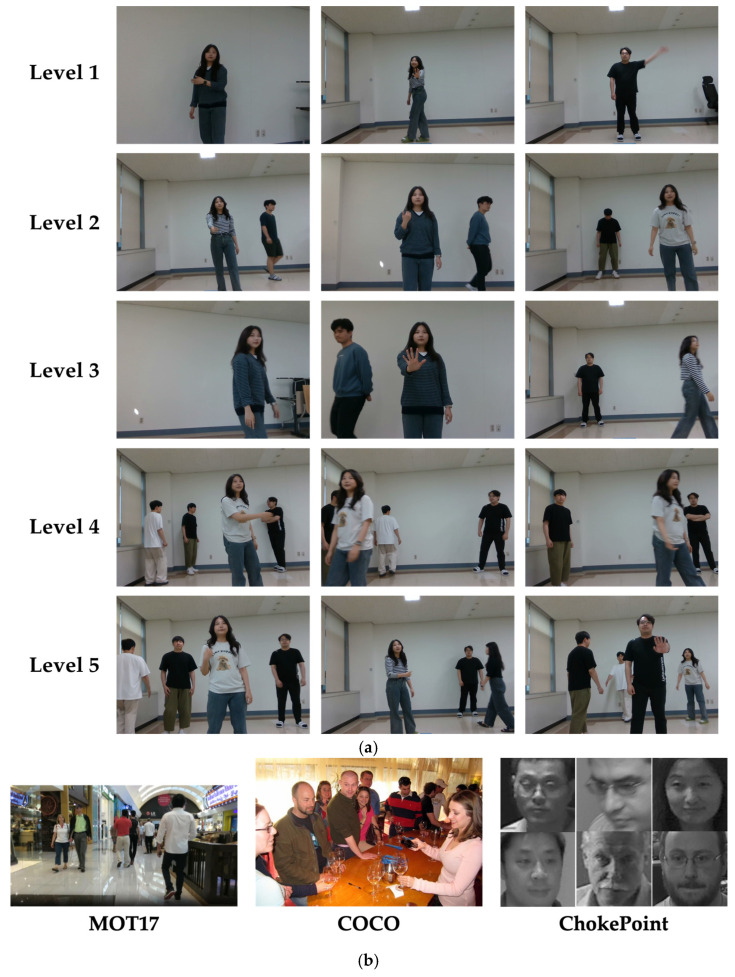
Dataset examples: (**a**) customized dataset, (**b**) benchmark datasets (MOT17, COCO, ChokePoint).

The evaluation of RFIS is comprised of two phases, one for evaluating individual components including person/body-part detection, person tracking, caretaker recognition and re-identification, and gesture recognition, and the other for evaluating RFIS as an integrated system. To this end, we custom-collected 24 video datasets in a real laboratory environment under five growing complexity levels of scenarios (refer to Section 7.5) with four, three, three, eight, and six video datasets allocated to the respective five levels, respectively. The average duration of each dataset was approximately six minutes. A video scene covering a distance ranging from 0.9 m to 5 m was taken by a camera located at about 1 m height from the ground, where the camera height was chosen to take account of the height of the hospital trolley. The customized datasets include the scenario in which both the caretaker and the non-caretakers are free to move around in the camera’s field of view. Out of 24 custom-collected video datasets, 18 were used for training and testing the individual components while six were saved exclusively for testing RFIS as an integrated system based on one, one, one, one, and two from the respective five levels. In terms of evaluating the individual components with 18 video datasets, the remaining three, two, two, seven, and four datasets after saving six datasets for testing RFIS were individually divided by an 8:2 ratio for training and testing. One exception is that the person/body-part detector was trained based on the MOT17 [40] and COCO [41] datasets together with the customized datasets. Figure 7 illustrates typical instances of the five complexity levels of custom-collected video datasets (a) and the benchmark datasets, MOT17, COCO, and ChokePoint (b), used for person/body-part detection.

### 7.1. Experiment of Person/Body-Part Detection and Classification

To evaluate the performance of the person/body-part detection and classification network, we utilized the MOT17 [40] and COCO [41] benchmark datasets along with a customized dataset collected in a real environment for training and testing. Specifically, the evaluation of the person detection incorporated 62,000 images from MOT17 and COCO for training, while 8300 indoor scene images from MOT17 and COCO, along with 200 images from the customized dataset, were used for testing. Meanwhile, the evaluation of the body-part detection employed 23,800 images from MOT by MOT17 and COCO along with 3000 images from the customized dataset for training, while 3570 images from MOT17 and COCO, alongside 1500 images from the customized dataset, were used for testing. The body-part classifier, attached to the YOLO detector and based on transfer learning, used 200 images from the customized dataset to refine the accuracy of body-part labeling under realistic scenarios.

**Figure 8:**
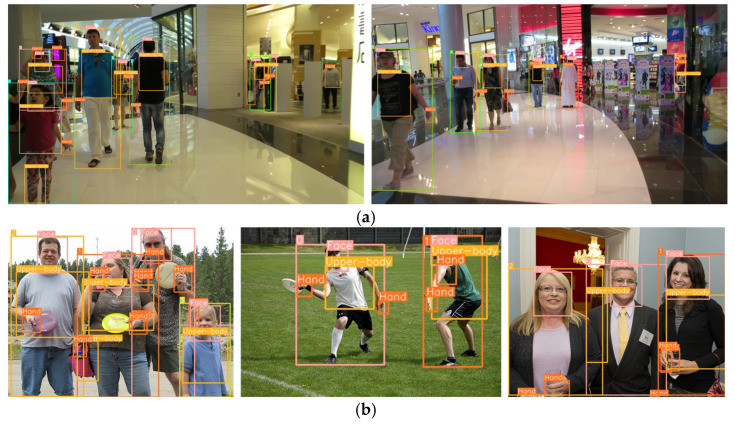

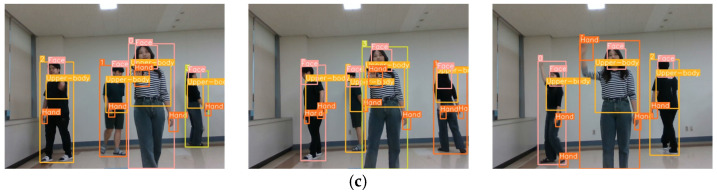
Person/body-parts detection and classification results of each dataset: (**a**) MOT17, (**b**) COCO, (**c**) customized dataset.

We used MODA (Multiple Object Detection Accuracy) and MODP (Multiple Object Detection Precision) as performance evaluation metrics [42] for evaluating the person/body-part detection and classification, as outlined in Equations (2) and (3).

(2) $$MODA\left(t\right)=1-\frac{c_{m}\left(m_{t}\right)+c_{f}(fp_{t})}{N_{G}^{t}}$$

(3) $$MODP\left(t\right)=\frac{\left(Mapped\;Overlap\;Ratio\right)}{N_{mapped}^{\left(t\right)}}$$

where $c_{m}$ and $c_{f}$ represent the cost function for missed detections and the penalty for false detections, respectively, while $N_{G}^{t}$ and $N_{mapped}^{\left(t\right)}$ represent the number of ground truth objects and the number of mapped objects, respectively, in the *t*th frame. The results, summarized in Table 1, show that the IoU threshold of 0.6 was employed to measure the MODA and MODP performance. It is important to note that the proposed transfer learning-based refinement significantly enhanced the MODA and MODP performances for body-part detection from 87% and 85.7% to 96.63% and 97.34%, respectively. Figure 8 shows the person and body-part detection results of (a) MOT17, (b) COCO, and (c) customized dataset.

### 7.2. Experiment of Person Tracking

**Figure 9:**
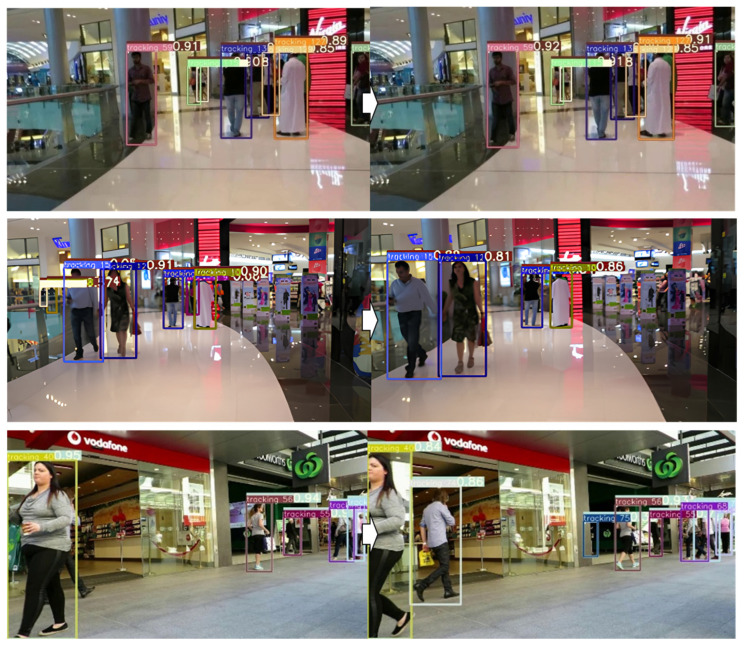
Person tracking results on the MOT17 benchmark dataset.

The proposed person-tracking algorithm was evaluated using 3000 image frames from the MOT17 dataset alongside 200 image frames from a specialized custom dataset, both of which were previously utilized for testing person detection and classification. We employed the MOTA (Multiple Object Tracking Accuracy) [42] metric to assess the tracking performance, as outlined in Equation (4), and the results are summarized in Table 2.

(4) $$MOTA=1-\frac{\sum_{i=1}^{N_{frames}}(c_{m}\left(m_{i}\right)+c_{f}\left(fp_{i}\right)+\log_{e}\left(ID_{switches})\right)}{\sum_{i=1}^{N_{frames}}N_{G}^{i}}$$

where $ID_{switches}$ represents the number of objects the tracking ID of which are switched along the procession of frames. Noteworthy is that the performance depicted in Table 2, which ranked 10 on the MOT17 leaderboard, includes contributions from the custom dataset sourced from a distinct environment in addition to the benchmark dataset. Figure 9 illustrates the typical results of person tracking in the MOT17 dataset. The same person is indicated with the same color over all the frames. The MOT17 dataset includes very complex scenes such as shopping malls, streets, or station halls, in which the person’s image size is too small to detect. However, the proposed tracking approach successfully tracked even if the box size was small.

**Figure 10:**
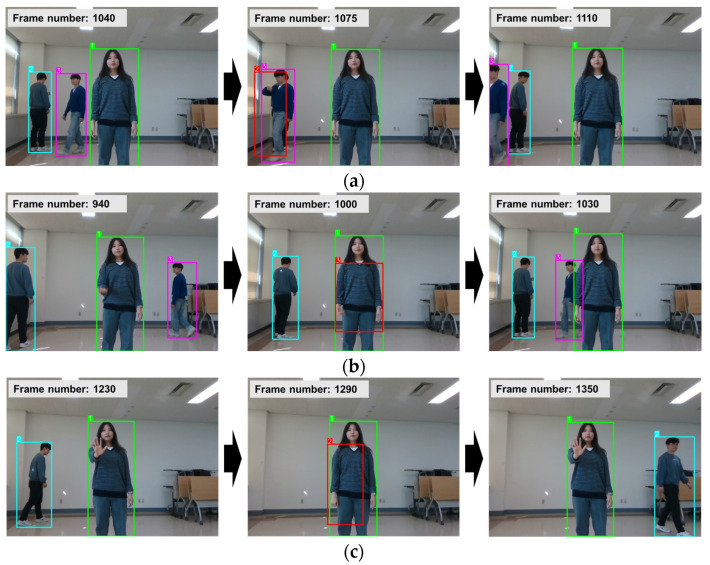
Typical instances of person detection, classification, and tracking. (**a**–**c**) are intended to illustrate the robustness of the proposed person tracking under ill-conditioned situations: (**a**) tracking in a narrow passage with overlapping, (**b**) tracking in a short-term interval of 2 s, and (**c**) tracking under temporary disappearance with crossing.

Importantly, while implementing the person tracking, we concentrated on optimizing computational efficiency to be well suited for an edge computing environment, achieving approximately 40 ms tracking time on the Jetson AGX Xavier, and simultaneously preserving a satisfactory level of tracking accuracy. Figure 10 demonstrates the performance of our integrated person-tracking, detection, and classification network. To illustrate the robustness of the person tracking, we selected three challenging scenarios: overlapping, disappearance, and reappearance of a person: (a) tracking in a narrow passage with overlapping, (b) tracking during a brief 2 s interval, and (c) tracking during temporary disappearance with crossing. In Figure 10, consistency is maintained by using the same color to represent the same ID of a person detected across the frames. It is crucial to note that our proposed person tracking dependably followed the person irrespective of these challenging conditions.

### 7.3. Experiment of Caretaker Recognition and Re-Identification

The performance of the proposed caretaker recognition system was assessed using two datasets: the ChokePoint dataset [43] as a benchmark and a custom dataset gathered in a real-world setting. The ChokePoint dataset contains 62,833 facial images from 29 persons, including 737 test images, 277 of which identify a caretaker. Out of 29 people, we randomly selected one person as a caretaker and the remaining 28 as non-caretakers. The custom dataset was compiled in crowded hallways and laboratories under varied lighting conditions, with some individuals wearing masks that partially occluded their faces. This dataset comprises 61,510 facial images from five persons including the caretaker, segmented into 44,058 training images and 17,452 testing images, with 10,369 and 4392 of those images, respectively, identified as caretaker images. Two of the five individuals are designated as caretakers, and the data for each caretaker consists of the following: Caretaker 1 (train: 15,278, test: 7277) and Caretaker 2 (train: 11,545, test: 3092). 

**Figure 11:**
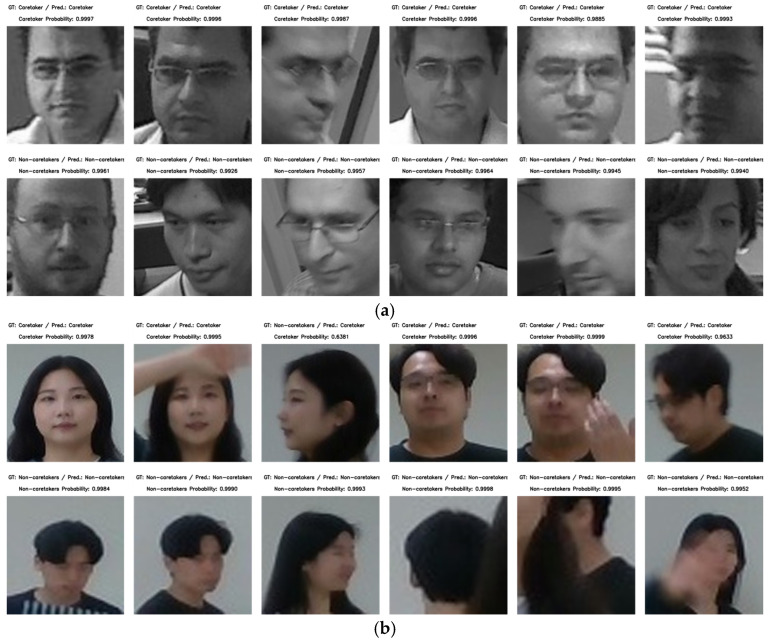
Caretaker recognition results of each dataset: (**a**) ChokePoint, (**b**) customized dataset. Note that the cropped face image is small because the face is a very small area of the overall scene image.

Figure 11 shows the caretaker recognition results of the ChokePoint dataset and the customized dataset. Figure 11a presents the prediction results for the ChokePoint dataset, where the first row depicts the prediction result for a caretaker, while the second row represents the result for the non-caretakers. The recognition accuracy is satisfactory for facial images captured from slightly skewed angles as well as when the caretaker is looking straight ahead. Figure 11b illustrates the results for the customized dataset. The first row depicts the prediction results for the two caretakers, while the second row shows the results for the remaining non-caretakers. The caretaker is recognized as a caretaker even when part of the face is obscured by gestures or the image is taken from the side of the face while walking. Conversely, the non-caretaker case encompasses not only the faces of non-caretakers but also the cases where the face is unidentifiable, such as the back of the head or where it is heavily occluded by other individuals.

The caretaker recognition accuracy was gauged using the pAUC metric [44], set to *p*-values of 1.0, 0.5, and 0.1, respectively. The results, summarized in Table 3, demonstrate that caretaker recognition achieves high accuracy, exceeding 95 pAUC at the *p*-value of 0.1 for the custom test dataset. 

As described, caretaker re-identification is based on the SSIM of face and upper-body box images between the caretaker and a candidate under evaluation. Table 4 illustrates that a notable disparity exists in the SSIM values of face and upper-body images in case the two persons at T − n and T frames are different, compared to the SSIM values in case the persons at T − n and T frames are the same. Based on the SSIM values of the face and upper-body box images between the two persons at T − n and T frames, the conditional probability, Equation (1), of the candidate box at T frame being the caretaker box at T − n frame, is shown in Table 4. We evaluated the performance of caretaker re-identification by using the three customized video datasets of level 3 complexity as testing datasets which consist of a total of 11 re-identification scenarios. We initiated the caretaker re-identification after the caretaker disappeared from the scene at least for 4 s. In the total 11 caretaker disappearance and appearance scenarios, there were 29 incidents of candidate evaluations, including 11 reappearances of the caretaker and 18 appearances of the non-caretaker. We set the threshold of maximum conditional probability as 0.75 to determine whether or not a candidate is the caretaker. In addition, to ensure the robustness of the decision, we introduced the indecision category to represent the case where either decision is too risky to take due to uncertainty. Specifically, indecision occurs in case the respective maximum and average conditional probabilities are either greater than 0.75 and less than 0.6 or less than 0.75 and greater than 0.65. In the case of indecision due to uncertainty, the decision is deferred to the subsequent frames. The performance of the proposed re-identification is summarized in Table 5. As shown, out of 29 incidents of re-identification, we obtained 11 correct decisions for 11 caretaker reappearances while 16 correct decisions, one incorrect decision, and one indecision for 18 non-caretaker appearances, resulting in 96.43% of accuracy.

One thing to note is that the proposed re-identification approach was intended to maximize computational efficiency with the SSIM-based conditional probabilities applied to a pair of detected face and upper-body image boxes, such that the re-identification process imposes little burden on implementing real-time RFIS in an edge computing environment. This contrasts with most of the state-of-the-art re-identification approaches in the literature, for instance, based on deep learning. As far as the benchmark performance is concerned, Dense Interaction Learning (DIL) proposed by He et al. [27] is currently top-ranked with 97.1 mAP for the DukeMTMC-reID [45] dataset and second-ranked with 87 mAP for the Motion Analysis and Re-identification (MARS) [46] dataset. However, DIL takes about 400 ms for process re-identification, compared to 40 ms of processing time by the proposed SSIM-based re-identification.

**Figure 12:**
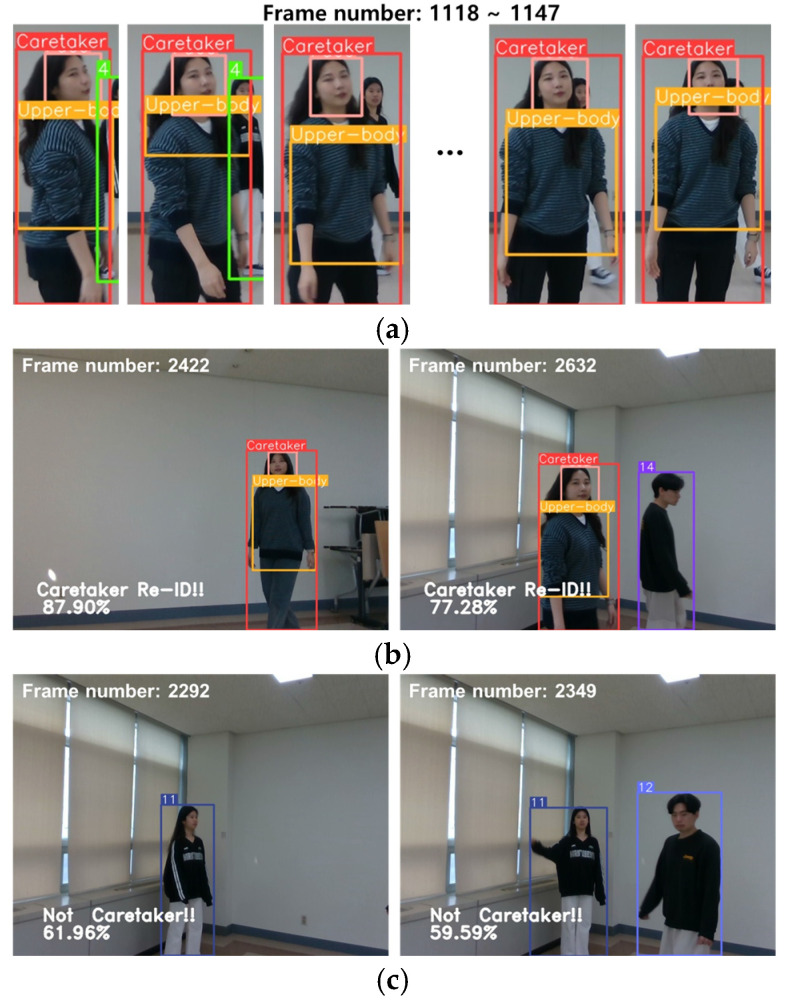
Various typical instances that occurred during the process of caretaker re-identification, where (**a**) shows the head and upper-body images of a caretaker, (**b**) illustrates the re-identification of a newly appeared candidate as a caretaker, and (**c**) illustrates the re-identification of a newly appeared candidate as a non-caretaker.

Figure 12 depicts various typical instances that occurred during the process of caretaker re-identification. Panel (a) shows the head and upper-body images of a caretaker, panel (b) illustrates the re-identification of a newly appeared candidate as a caretaker, and panel (c) shows the re-identification of a newly appeared candidate as a non-caretaker. It is important to note, as discussed in Section 5, that the decision regarding caretaker re-identification is conclusively made based on additional evidence gathered over the subsequent 40 frames following the initial re-identification.

### 7.4. Experiment of Gesture Recognition

**Figure 13:**
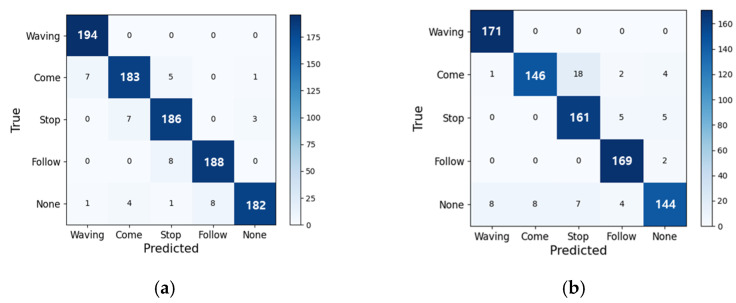
The confusion matrix detailing the performance of proposed gesture recognition for the five categories of gestures: (**a**) the results of level 1 to 4 scenarios. (**b**) the results of the level 5 scenario.

For the evaluation of caretaker gesture recognition, we used 18 customized video datasets of up to level 4 complexity for training and six customized video datasets of up to level 5 complexity for testing. Note that the six video datasets for testing are the same saved for testing RFIS as an integrated system. The 18 customized video datasets for training amount to a total of 4000 gesture sequences of five gesture categories: “Here I am (Waving)”, “Come here (Come)”, “Stay there (Stop)”, “Follow me (Follow)”, and “None”. Each gesture sequence consists of 2 s or 120 frames of video images. For testing with the six customized video datasets, we tested the two cases separately: (1) testing a total of 980 gesture sequences of up to the level 4 complexity with no alteration of the caretaker and (2) testing a total of 855 gesture sequences of the level 5 complexity with two caretakers alternated. Table 6 shows the accuracy of gesture recognition associated with five gesture categories for the above two cases. As shown, we obtained the average gesture recognition accuracy of 93.9% by averaging over all five gesture categories from both cases. Figure 13 provides confusion matrices showing performance details. Figure 13 indicates that the relatively lower accuracy associated with the “Come here” gesture is from its confusion with the “Stay there” gesture as the two have a common hand trajectory in a large part of their motions. Gesture recognition in RFIS is carried out under continuous interactions with the intent of gesture properly delivered, such that there exists a high probability of correcting the error, if necessary, in the subsequent interactions. We compared the performance of the proposed gesture recognition with the state-of-the-art performance [33] based on the action recognition benchmark datasets, in particular, the action recognition UTD-MHAD [34] dataset. We selected [33] for comparison since a part of the 27 actions used in [33] are similar to our gestures while its action recognition is based on RGB images. Ref. [33] obtained 92.5% accuracy for 27 action classes while we obtained 93.9% accuracy for five gesture classes. However, they considered only the simplest action scenario with a single actor, representing level 1 complexity, whereas we considered up to level 5 complexity in evaluation scenarios.

**Figure 14:**
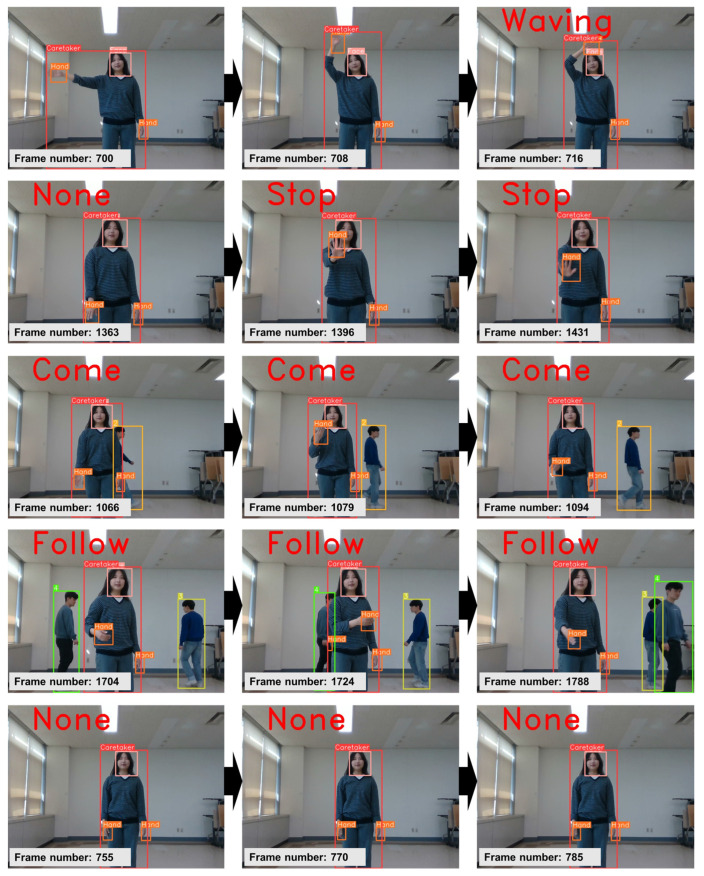
Typical instances of 5 categories of gestures recognized in real time within an authentic environment are showcased, utilizing the gesture recognition network integrated into RFIS. Note: the use of a low-resolution camera for computational efficiency somewhat compromises the image quality.

Figure 14 displays typical instances of five categories of gestures recognized in real time within a realistic setting, employing the proposed gesture recognition network integrated into RFIS, with each caretaker highlighted in a red box.

### 7.5. Experiment of RFIS as an Integrated System

**Figure 15:**
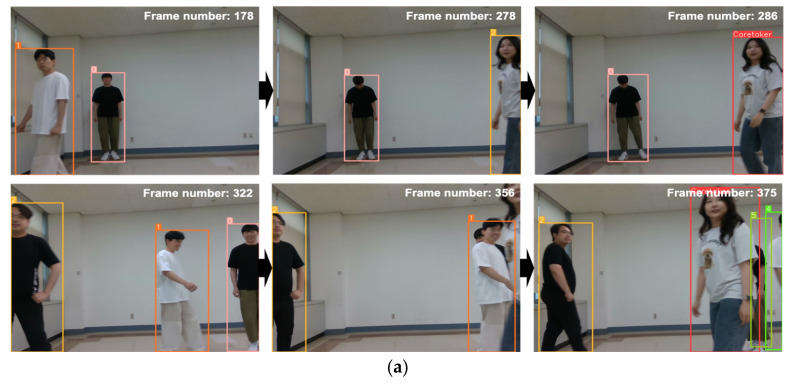

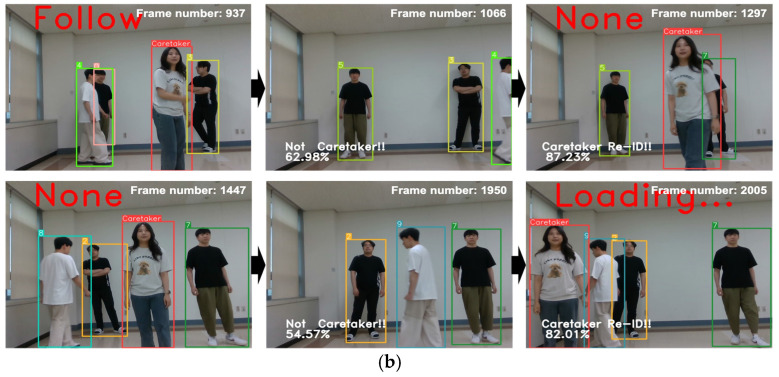
The caretaker recognition and re-identification results of the level 4 scenario experiment: (**a**) the caretaker recognition. (**b**) the caretaker re-identification.

**Figure 16:**
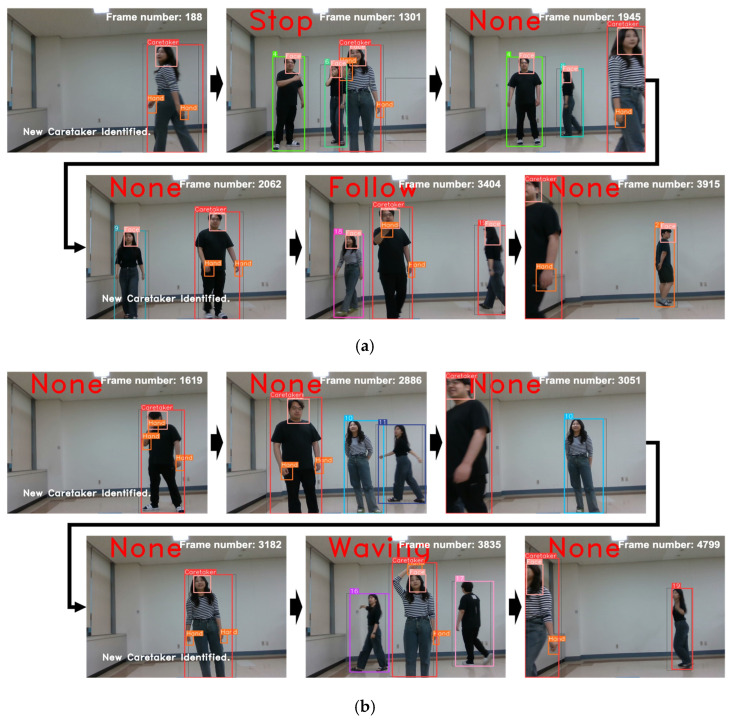
The caretaker recognition and re-identification results of the level 5 scenario experiment: (**a**) the female caretaker recognition and switched to male caretaker by recognition after the female caretaker’s disappearance. (**b**) the male caretaker recognition and switched to female caretaker by recognition after the male caretaker’s disappearance.

To assess the overall quality of interaction facilitated by RFIS as an integrated system, we custom-collected five video scenarios independently of the datasets used for training and testing the individual components of RFIS. Each video scenario intends to represent a different level of interaction complexity including challenging conditions. For the five scenarios of different interaction complexity, a total of 26 video datasets were collected in a real laboratory environment. The five video scenarios consist of the following five levels of complexity: (1) a single caretaker present in a scene (1 C + 0 N/C), (2) a single caretaker plus up to two non-caretakers present in a scene (1 C + 2 N/C), (3) a single caretaker plus up to two non-caretakers present in a scene with the caretaker disappearing and reappearing (1 C + 2 N/C + ReID), (4) a single caretaker plus up to three non-caretakers present in a scene with the caretaker disappearing and reappearing (1 C + 3 N/C + ReID), (5) two caretakers plus up to three non-caretakers present in a scene with the caretakers disappearing and reappearing (2 C + 3 N/C + ReID). Each of the five levels of scenarios involves caretaker recognition, tracking, and gesture recognition whereas only levels 3, 4, and 5 perform caretaker re-identification. Note that levels 4 and 5 represent the most complex interaction scenarios with the highest crowdedness and multiple caretaker protocols. Figure 15 shows typical results of the caretaker recognition, re-identification, tracking, and gesture recognition in level 4, where (a) and (b) show the results of caretaker recognition and re-identification, respectively, in a crowded environment. Notice in Figure 15b that, in the situation of the caretaker’s disappearance and reappearance, RFIS can accurately recognize the non-caretakers and re-identify the caretaker from a newly appeared person along the lapse of video frames. Level 5 is distinct from the preceding levels in that two caretakers are simultaneously present in a scene. In that case, we set the following rule for caretaker identification from the scene: (1) the caretaker identified in the current scene is given priority as long as he/she remains in the scene, (2) in case the current caretaker disappears from a scene and does not reappear in a certain amount of time, then one of the multiple caretakers who appears first in a scene is designated as the caretaker. Figure 16 illustrates a level 5 scenario, where (a) RFIS keeps track of the female caretaker (red box) identified in the first place even though a male caretaker (green box) walks into the scene. Figure 16b illustrates the case where RFIS identifies a female caretaker who appeared first in the scene after the disappearance of the male caretaker as a caretaker.

Table 7 presents the overall performance of RFIS summarized in terms of five complexity levels of scenarios as a fully integrated system. In Table 7, the accuracies of RFIS in caretaker recognition, tracking, re-identification, and gesture recognition are compared across the five scenarios of varying complexity. In Table 7, “C”, “N/C”, and “ReID” represent caretaker, non-caretaker, and re-identification, respectively. Table 7 indicates that the proposed RFIS can provide sufficiently high accuracy for caretaker recognition, tracking, re-identification, and gesture recognition despite the complexity level of scenarios moving up to the highest. Note that the accuracy associated with caretaker tracking is purely based on the motion-based prediction of a caretaker box without fusing the result from caretaker recognition. For additional details, refer to the videos corresponding to the five levels of complexity of the scenarios, available at the following link: https://youtu.be/nKncFBXBGAw (accessed on 11 July 2024).

### 7.6. Discussion

The performance of RFIS, as summarized in Table 7, is considered sufficient to effectively facilitate HRI, achieving an average accuracy of over 90% as an integrated system for caretaker recognition, tracking, re-identification, and gesture recognition, while maintaining a real-time operation of 5 FPS. Nonetheless, we suggest that further experiments involving the developed RFIS would be advantageous, particularly to address unexpected situations, including novel and unconventional HRI scenarios that might emerge in more complex interaction environments.

## 8. Conclusions

A Robot-Facilitated Interaction System (RFIS) has been designed and implemented to enable proactive facilitation by an autonomous mobile robot transporting a patient for real-time, user-friendly interaction with a caretaker who monitors and nurses the patient. The proposed RFIS integrates the detection, classification, and tracking of persons/body parts with caretaker recognition, re-identification, and gesture recognition modules into a cohesive real-time system operational in an edge computing environment. This paper demonstrates that by effectively integrating deep learning with traditional engineering approaches, a complex HRI facilitation system comprising several functional modules of high complexity can be achieved in a real-time system with state-of-the-art performance in terms of overall accuracy and an operational speed of approximately 5 FPS. For instance, we achieved over 90% in MODA and MODP and over 70% in MOTA metrics based on the custom-collected testing datasets, which may be indirectly compared with the current state-of-the-art performance of 50–90% for MODA [47] and MODP [47] and 40–70% for MOTA [47] (Note: This indirect comparison is noted due to differences in the testing environments.) In particular, we were able to assess the overall interaction quality of RFIS, performing caretaker recognition, tracking, re-identification, and gesture recognition as a fully integrated system, and found that the RFIS is capable of providing effective HRI facilitation with over 90% accuracies on average in a real-time operation of 5 FPS.

For effective integration, we devised several novel approaches, including improving the accuracy of body-part labeling while maintaining a high level of detection recall, employing Bayesian conditional probabilities associated with predicted and detected boxes for person and body-part tracking, and directly linking person and body-part tracking to caretaker re-identification and gesture recognition. In the future, we plan to expand our experiments to a variety of real-world settings with more complex interaction scenarios to further enhance performance, while continuing to refine RFIS as an integrated system.

In the future, we plan to expand our experiments to more varieties of real-world settings including a hospital environment, possibly, with a higher complexity level of interaction scenarios, such that we can find many challenging conditions to be addressed for RFIS. In addition, we plan to continue searching for alternative approaches to caretaker detection, recognition, tracking, re-identification, and gesture recognition, whether custom-designed or adopted from open-source platforms, that can provide better performance for real-time RFIS operations in real-world settings. For instance, we plan to explore how the recent advancement of YOLO versions from v7 to v10, in particular, YOLOv8, provides a new opportunity to further improve the performance of person/body-part detection and, consequently, of the overall performance of RFIS as an integrated system.

## Figures and Tables

**Table 1:** MODA and MODP performance of the proposed person/body-part detection and classification.

	IoU threshold	MODA	MODP
Person Detection	0.6	94.7%	94.6%
Body-part Detection	0.6	96.63%	97.34%

**Table 2:** Performance of the proposed person/body-part tracking evaluated by the MOTA metric.

Tracking	MOTA
Person Tracking	74.96%

**Table 3:** pAUC performance of the proposed caretaker recognition.

	*p*-Value	pAUC
ChokePoint Dataset	1.0	1.0
	0.5	1.0
	0.1	1.0
Customized Dataset—Single Caretaker	1.0	0.9965
	0.5	0.9955
	0.1	0.9835
Customized Dataset—Multiple Caretaker	1.0	0.9958
	0.5	0.9948
	0.1	0.9829

**Table 4:** Typical SSIM values of face and upper-body images at T − n and T frames for Person A and Person B.

	T Frame	Person A	Person B
T − n Frame		(Caretaker)	(Non-Caretaker)
Person A (Caretaker)	Face	0.66830	0.05613
	Upper-Body	0.41907	0.13146
	Conditional Probability	0.5283	0.0286
Person B (Non-Caretaker)	Face	0.08085	0.73129
	Upper-Body	0.11232	0.69456
	Conditional Probability	0.0350	0.6701

**Table 5:** The results of accuracy, recall, and precision were evaluated with 29 caretaker re-identification situations in terms of caretaker and non-caretaker predictions.

	GT	Caretaker	Non-Caretaker	Recall	Precision	Accuracy
Prediction						
Caretaker		11	1	1.0	0.9167	0.9643
Non-Caretaker		0	16	0.9412	1.0	

DIL [27]: 97.1 mAP (DukeMTMC-reID), 87 mAP (MARS).

**Table 6:** Accuracy of the proposed gesture recognition for the five categories of gestures.

Gesture Type	Accuracy		
	Case 1 (Single)	Case 2 (Multiple)	
“Here I am”	100%	100%	Average 93.9%
“Stay there”	94.8%	94.1%	
“Come here”	93.3%	85.3%	
“Follow me”	95.9%	98.8%	
“No gesture”	92.8%	84.2%	

Action machine [33] accuracy: 93.8%.

**Table 7:** The overall interaction quality of RFIS as a fully integrated system, where the accuracies of RFIS in caretaker recognition, tracking, re-identification, and gesture recognition are shown for the three different complexity scenarios.

			1 C + 0 N/C	1 C + 2 N/C	1 C + 2 N/C + ReID	1 C + 3 N/C + ReID	2 C + 3 N/C + ReID
Caretaker Recognition	Accuracy		100%	100%	100%	100%	100%
Caretaker Tracking	MOTA		100%	100%	87.5% (1 failure)	100%	100%
Caretaker Re-identification	Accuracy		N/A	N/A	100%	100%	93.75% (1 failure)
Caretaker Gesture Recognition	Accuracy	“Here I am”	95.8%	100%	95.8%	98.7%	100%
		“Stay there”	95.6%	100%	89.2%	91.8%	90.6%
		“Come here”	89.2%	86.3%	80.0%	92.2%	85.6%
		“Follow me”	96.1%	97.1%	100%	94.6%	95.3%
		None	97.4%	100%	88.1%	92.9%	86.4%

## Data Availability

The data presented in this study are available on request from the corresponding authors due to their containing personal facial information.

## References

[1] Lee I. (2021). Service robots: A systematic literature review. Electronics.

[2] Lee S., Lee S., Kim S., Kim A. (2023). Robot-Facilitated Human–Robot Interaction with Integrated Tracking, Re-identification and Gesture Recognition. Proceedings of the International Conference on Intelligent Autonomous Systems.

[3] Sanjeewa E.D.G., Herath K.K.L., Madhusanka B.G.D.A., Priyankara H.D.N.S. (2020). Visual attention model for mobile robot navigation in domestic environment. GSJ.

[4] Zhao X., Naguib A.M., Lee S. (2014). Kinect based calling gesture recognition for taking order service of elderly care robot. Proceedings of the 23rd IEEE International Symposium on Robot and Human Interactive Communication.

[5] Liu C., Szirányi T. (2021). Real-time human detection and gesture recognition for on-board UAV rescue. Sensors.

[6] Rollo F., Zunino A., Raiola G., Amadio F., Ajoudani A., Tsagarakis N. (2023). Followme: A robust person following framework based on visual re-identification and gestures. Proceedings of the 2023 IEEE International Conference on Advanced Robotics and Its Social Impacts (ARSO).

[7] Müller S., Wengefeld T., Trinh T.Q., Aganian D., Eisenbach M., Gross H.-M. (2020). A multi-modal person perception framework for socially interactive mobile service robots. Sensors.

[8] Ren S., He K., Girshick R., Sun J. Faster r-cnn: Towards real-time object detection with region proposal networks. Proceedings of the Advances in Neural Information Processing Systems.

[9] Redmon J., Divvala S., Girshick R., Farhadi A. You only look once: Unified, real-time object detection. Proceedings of the IEEE Conference on Computer Vision and Pattern Recognition.

[10] Liu W., Anguelov D., Erhan D., Szegedy C., Reed S., Fu C.Y., Berg A.C. SSD: Single shot multibox detector. Proceedings of the European Conference on Computer Vision (ECCV).

[11] Zhang H., Li F., Liu S., Zhang L., Su H., Zhu J., Ni L.M., Shum H.Y. (2022). Dino: Detr with improved denoising anchor boxes for end-to-end object detection. arXiv.

[12] Liu Z., Hu H., Lin Y., Yao Z., Xie Z., Wei Y., Ning J., Cao Y., Zhang Z., Dong L. Swin transformer v2: Scaling up capacity and resolution. Proceedings of the IEEE/CVF Conference on Computer Vision and Pattern Recognition.

[13] Qiao S., Chen L.C., Yuille A. Detectors: Detecting objects with recursive feature pyramid and switchable atrous convolution. Proceedings of the IEEE/CVF Conference on Computer Vision and Pattern Recognition.

[14] Lee T.-H., Kim K.-J., Yun K.-S., Kim K.-J., Choi D.-H. (2018). A method of Counting Vehicle and Pedestrian using Deep Learning based on CCTV. J. Korean Inst. Intell. Syst..

[15] Mukhtar A., Cree M.J., Scott J.B., Streeter L. (2018). Mobility aids detection using convolution neural network (cnn). Proceedings of the 2018 International Conference on Image and Vision Computing New Zealand (IVCNZ).

[16] Fernando T., Denman S., Sridharan S., Fookes C. (2018). Tracking by prediction: A deep generative model for mutli-person localisation and tracking. Proceedings of the 2018 IEEE Winter Conference on Applications of Computer Vision (WACV).

[17] Choi W. Near-online multi-target tracking with aggregated local flow descriptor. Proceedings of the IEEE International Conference on Computer Vision.

[18] Manzoor S., Kim E.-J., Bae S.-H., Kuc T.-Y. (2023). Edge Deployment of Vision-Based Model for Human Following Robot. Proceedings of the 2023 23rd International Conference on Control, Automation and Systems (ICCAS).

[19] Jader G., Fontineli J., Ruiz M., Abdalla K., Pithon M., Oliveira L. (2018). Deep face recognition: A survey. Proceedings of the 2018 31st SIBGRAPI Conference on Graphics, Patterns and Images (SIBGRAPI).

[20] Sohail M., Shoukat I.A., Khan A.U., Fatima H., Jafri M.R., Yaqub M.A., Liotta A. (2024). Deep Learning Based Multi Pose Human Face Matching System. IEEE Access.

[21] Condés I., Fernández-Conde J., Perdices E., Cañas J.M. (2023). Robust Person Identification and Following in a Mobile Robot Based on Deep Learning and Optical Tracking. Electronics.

[22] Schroff F., Kalenichenko D., Philbin J. Facenet: A unified embedding for face recognition and clustering. Proceedings of the IEEE Conference on Computer Vision and Pattern Recognition.

[23] Ye M., Shen J., Lin G., Xiang T., Shao L., Hoi S.C. (2021). Deep learning for person re-identification: A survey and outlook. IEEE Trans. Pattern Anal. Mach. Intell..

[24] Wei W., Yang W., Zuo E., Qian Y., Wang L. (2022). Person re-identification based on deep learning—An overview. J. Vis. Commun. Image Represent..

[25] Wang G., Lai J., Huang P., Xie X. Spatial-temporal person re-identification. Proceedings of the AAAI Conference on Artificial Intelligence.

[26] Rollo F., Zunino A., Tsagarakis N., Hoffman E.M., Ajoudani A. (2013). Carpe-id: Continuously adaptable re-identification for personalized robot assistance. arXiv.

[27] He T., Jin X., Shen X., Huang J., Chen Z., Hua X.S. Dense interaction learning for video-based person re-identification. Proceedings of the IEEE/CVF International Conference on Computer Vision.

[28] Narayana P., Ross B., Draper B.A. Gesture recognition: Focus on the hands. Proceedings of the IEEE Conference on Computer Vision and Pattern Recognition.

[29] Zhang L., Zhu G., Shen P., Song J., Shah S.A., Bennamoun M. Learning spatiotemporal features using 3dcnn and convolutional lstm for gesture recognition. Proceedings of the IEEE International Conference on Computer Vision Workshops.

[30] Al-Hammadi M., Muhammad G., Abdul W., Alsulaiman M., Bencherif M.A., Mekhtiche M.A. (2020). Hand gesture recognition for sign language using 3DCNN. IEEE Access.

[31] Dadashzadeh A., Targhi A.T., Tahmasbi M., Mirmehdi M. (2019). HGR-Net: A fusion network for hand gesture segmenta-tion and recognition. IET Comput. Vis..

[32] Yu J., Qin M., Zhou S. (2022). Dynamic gesture recognition based on 2D convolutional neural network and feature fusion. Sci. Rep..

[33] Zhu J., Zou W., Xu L., Hu Y., Zhu Z., Chang M., Huang J., Huang G., Du D. (2018). Action machine: Rethinking action recognition in trimmed videos. arXiv.

[34] Chen C., Jafari R., Kehtarnavaz N. (2015). UTD-MHAD: A multimodal dataset for human action recognition utilizing a depth camera and a wearable inertial sensor. Proceedings of the 2015 IEEE International Conference on Image Processing (ICIP).

[35] Redmon J., Ali F. (2018). Yolov3: An incremental improvement. arXiv.

[36] Tan M., Le Q. (2019). Efficientnet: Rethinking model scaling for convolutional neural networks. Proceedings of the International Conference on Machine Learning.

[37] Nepal U., Eslamiat H. (2022). Comparing YOLOv3, YOLOv4 and YOLOv5 for autonomous landing spot detection in faulty UAVs. Sensors.

[38] Stanojevic V.D., Todorovic B.T. (2024). BoostTrack: Boosting the similarity measure and detection confidence for improved multiple object tracking. Mach. Vis. Appl..

[39] Hore A., Ziou D. (2010). Image quality metrics: PSNR vs. SSIM. Proceedings of the 2010 20th International Conference on Pattern Recognition.

[40] Milan A., Leal-Taixe L., Reid I., Roth S., Schindler K. (2016). MOT16: A benchmark for multi-object tracking. arXiv.

[41] Lin T.Y., Maire M., Belongie S., Hays J., Perona P., Ramanan D., Dollár P., Zitnick C.L. (2014). Microsoft Coco: Common Objects in context. Proceedings of the Computer Vision–ECCV 2014: 13th European Conference.

[42] Kasturi R., Goldgof D., Soundararajan P., Manohar V., Garofolo J., Bowers R., Boonstra M., Korzhova V., Zhang J. (2008). Framework for performance evaluation of face, text, and vehicle detection and tracking in video: Data, metrics, and protocol. IEEE Trans. Pattern Anal. Mach. Intell..

[43] Wong Y., Chen S., Mau S., Sanderson C., Lovell B.C. (2011). Patch-based probabilistic image quality assessment for face selection and improved video-based face recognition. Proceedings of the CVPR 2011 Workshops.

[44] Dodd L.E., Pepe M.S. (2003). Partial AUC estimation and regression. Biometrics.

[45] Ristani E., Solera F., Zou R., Rita C., Carlo T. (2016). Performance measures and a data set for multi-target, multi-camera tracking. Proceedings of the European Conference on Computer Vision.

[46] Zheng L., Bie Z., Sun Y., Wang J., Su C., Wang S., Tian Q. (2016). Mars: A video benchmark for large-scale person re-identification. Proceedings of the Computer Vision–ECCV 2016: 14th European Conference.

[47] Hyun J., Kang M., Wee D., Yeung D.Y. Detection recovery in online multi-object tracking with sparse graph tracker. Proceedings of the IEEE/CVF Winter Conference on Applications of Computer Vision.

